# Applications of Multivariate Statistical and Data Mining Analyses to the Search for Biomarkers of Sensorineural Hearing Loss, Tinnitus, and Vestibular Dysfunction

**DOI:** 10.3389/fneur.2021.627294

**Published:** 2021-03-03

**Authors:** Paul F. Smith, Yiwen Zheng

**Affiliations:** ^1^Department of Pharmacology and Toxicology, Brain Health Research Centre, School of Biomedical Sciences, University of Otago, Dunedin, New Zealand; ^2^Brain Research New Zealand Centre of Research Excellence, University of Auckland, Auckland, New Zealand; ^3^The Eisdell Moore Centre for Hearing and Balance Research, University of Auckland, Auckland, New Zealand

**Keywords:** multivariate statistical analysis, data mining, orthogonal partial least squares discriminant analysis, hearing loss, tinnitus, vestibular dysfunction

## Abstract

Disorders of sensory systems, as with most disorders of the nervous system, usually involve the interaction of multiple variables to cause some change, and yet often basic sensory neuroscience data are analyzed using univariate statistical analyses only. The exclusive use of univariate statistical procedures, analyzing one variable at a time, may limit the potential of studies to determine how interactions between variables may, as a network, determine a particular result. The use of multivariate statistical and data mining methods provides the opportunity to analyse many variables together, in order to appreciate how they may function as a system of interacting variables, and how this system or network may change as a result of sensory disorders such as sensorineural hearing loss, tinnitus or different types of vestibular dysfunction. Here we provide an overview of the potential applications of multivariate statistical and data mining techniques, such as principal component and factor analysis, cluster analysis, multiple linear regression, random forest regression, linear discriminant analysis, support vector machines, random forest classification, Bayesian classification, and orthogonal partial least squares discriminant analysis, to the study of auditory and vestibular dysfunction, with an emphasis on classification analytic methods that may be used in the search for biomarkers of disease.

## Introduction

**Figure 1:**
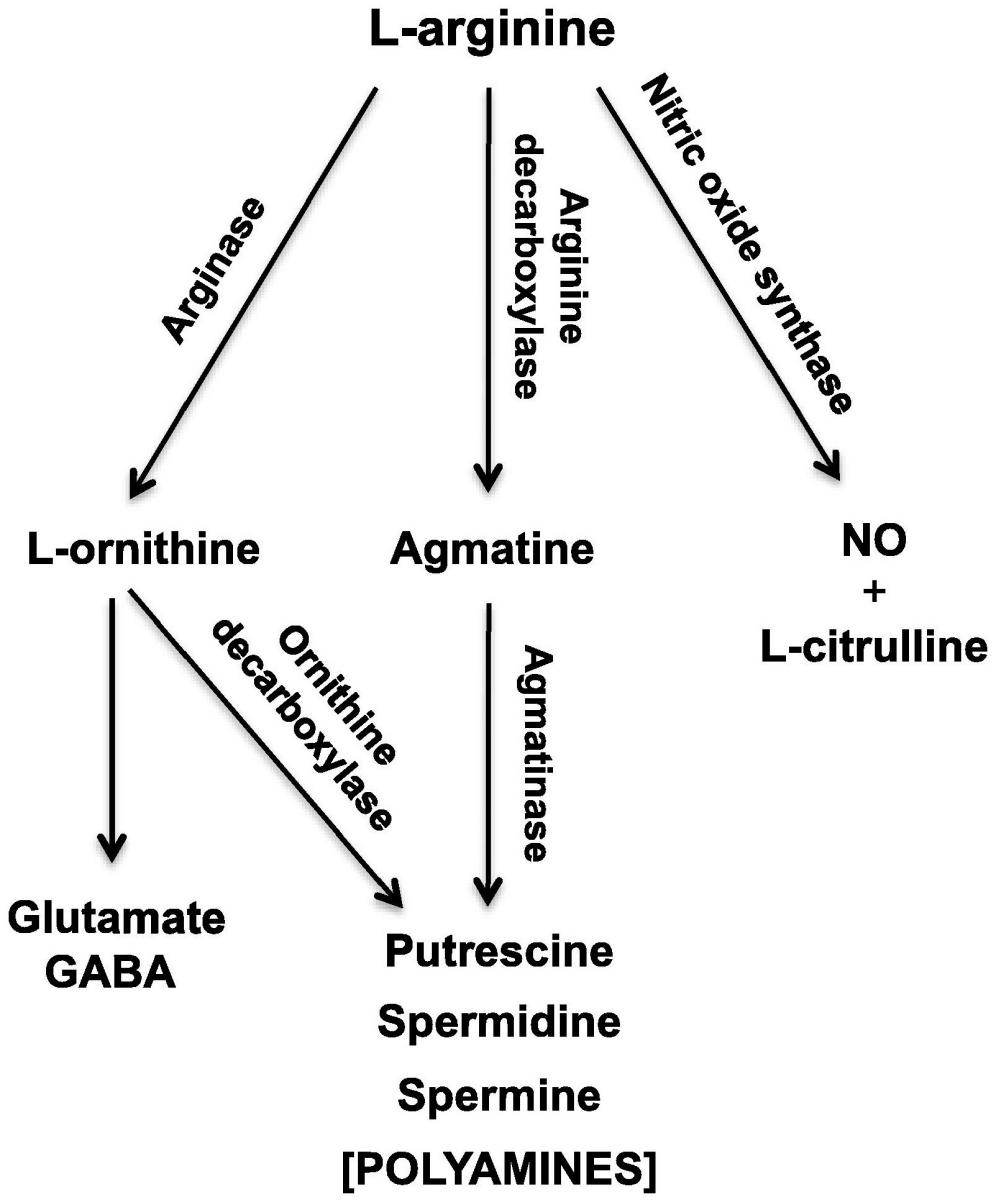
Schematic diagram of the L-arginine metabolic pathways in the vestibular nucleus and cerebellum, which change during the brain aging process. NO, nitric oxide; GABA, γ-aminobutyric acid. From Liu et al. (4) with permission.

**Figure 2:**
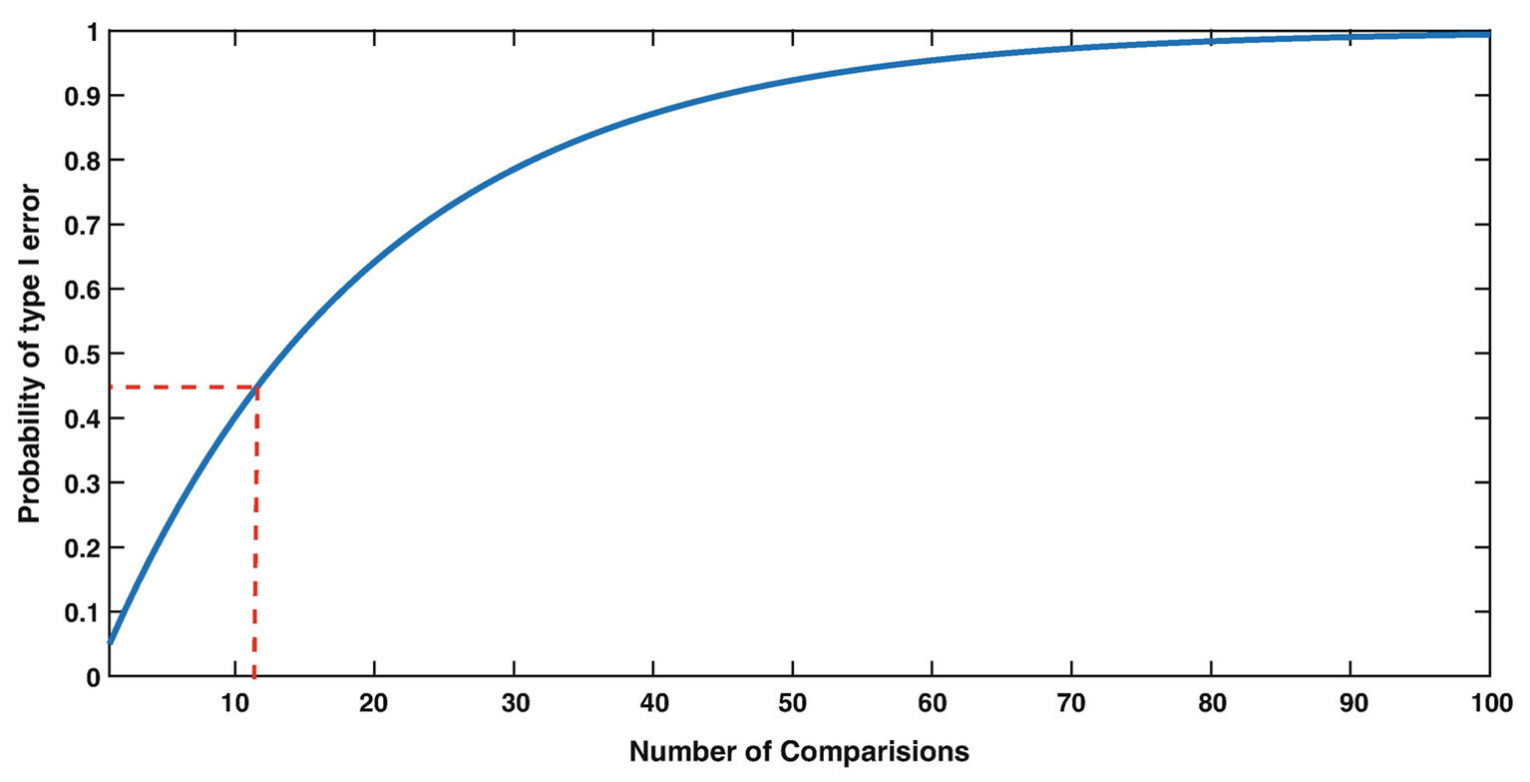
The Type I error rate (False Alarm rate) strongly depends on the number of comparisons. For example, with 12 comparisons (red dashed lines), the probability of making at least one False Alarm is 0.46, i.e., much increased compared to 0.05 with only 1 comparison. From Herzog et al. (7) with permission.

Experimental phenomena in neuroscience often involve the complex, sometimes non-linear interaction, of multiple variables. In the context of sensorineural hearing loss (SNHL), tinnitus or vestibular disorders, a number of independent variables may interact with one another, such as age, sex, drug use, and genetic predispositions; similarly, many biochemical systems may interact with one another to cause such disorders (see Figure 1 for an example in the context of age-related neurochemical changes in the brainstem vestibular nucleus and cerebellum). Despite this, the majority of statistical analyses in basic auditory and vestibular neuroscience have tended to focus on comparisons between treatment groups, analyzing one variable at a time. In many areas of sensory neuroscience in general, univariate statistical analyses have been used almost exclusively. This approach neglects the fact that changes may occur at the level of the interaction within a network or system of variables, that cannot be detected in any individual variable alone (1–6) (see Figure 1 for an example). In addition, the use of multiple univariate statistical analyses may inflate the type 1 error rate, or the probability of rejecting the null hypothesis when it is true, as a result of a large number of individual analyses (1, 2, 7) (Figure 2). In situations in which there are a large number of variables, for example, gene microarray, proteomic and metabolomic data, and more recently, medical diagnostics, multivariate statistical analyses and data mining approaches have been increasingly employed in order to understand the complex interactions that can occur between systems of variables, as well as to avoid increasing the type I error rate [e.g., (6, 8–30)].

Multivariate statistical analyses (MVAs) and data mining analyses can be broadly divided into those that are “supervised” and those that are “unsupervised”. A “supervised” method of analysis is directed at a specific dependent variable, in order to determine the relationship between a set of independent variables and one or more dependent variables, (e.g., to make a prediction; e.g., multiple linear regression). By contrast, in “unsupervised” methods, there is no specific dependent variable; instead, the objective is to explore associations between variables (e.g., cluster analyses) (see Table 1). Furthermore, some MVAs and data mining methods are concerned with predicting categorical variables (“classification,” e.g., linear discriminant analysis), and some concerned with predicting continuous variables (“regression,” e.g., multiple linear regression). Some of these methods involve only one dependent variable, e.g., multiple linear regression, while others may involve multiple dependent variables, e.g., canonical correlation analysis; however, for the purposes of this paper, MVAs will be defined as a collection of methods that involve *multiple variables*, either independent or dependent, or both. Clearly, regression methods such as multiple linear regression can be extended to include more than one dependent variable [e.g., multivariate multiple regression; (1, 2)].

**Table 1:** Different types of MVA and Data Mining Methods categorized according to whether they involve a categorical or continuous (quantitative) dependent variable and whether they specify a dependent variable (i.e., Supervised) or not (i.e., Unsupervised).

**Supervised**
**Qualitative or categorical variables**
Linear discriminant analysis Logistic regression Partial least squares discriminant analysis Structural equation modeling Support vector machines (DM) Random forest classification (DM) Neural networks (DM) K nearest neighbors
**Quantitative variables**
Multiple linear regression Canonical correlation analysis Multivariate multiple regression Structural equation modeling Random forest regression (DM) Gradient boosted decision trees (DM) Neural networks (DM) K nearest neighbors
**Unsupervised**
**Qualitative or categorical variables**
Correspondence analysis
**Quantitative variables**
Principal component analysis Factor analysis Cluster analysis Multidimensional scaling Ordination

*“DM” denotes those methods that emerged out of dating mining research in computer science. From Smith (31) with permission*.

Some unsupervised MVAs are not focussed on a specific dependent variable or the implication of causality, but more the degree of co-variation amongst multiple variables, as an indicator of association. For example, cluster analyses could be used to investigate the degree to which different variables related to SNHL co-vary with one another. Cluster analyses have been used extensively in genomics and proteomics research as a means of exploring the association between variables. Still other MVAs are concerned with investigating the way that groups of variables with different weightings, explain most of the variation in a system of variables (e.g., Figure 1; e.g., principal component analysis). Data mining analyses are related to MVAs; however, some have arisen out of computer science rather than conventional statistics. Data mining analyses include procedures such as random forest regression, random forest classification and support vector machines.

The aim of this paper is to provide a succinct guide to some MVAs and data mining analytic methods that can be applied to auditory and vestibular neuroscience data related to SNHL, tinnitus, and vestibular dysfunction, at both the basic experimental and clinical levels. Particular emphasis will be placed on “classification methods” that are relevant to the search for biomarkers of auditory and vestibular dysfunction, i.e., linear discriminant analysis, support vector machines, random forest classification, Bayesian classification, and orthogonal partial least squares discriminant analysis. However, classification and regression methods are related statistically, and for this reason, regression methods, in which there is no categorical dependent variable, will also be addressed. The review is intended to exemplify the application of MVA and data mining methods to problems in vestibular and auditory neuroscience, and is not meant to be exhaustive in terms of the specific methods described; procedures such as artificial neural network modeling (ANN), structural equation modeling, multivariate regression, canonical correlation analysis and many others, are also important, but are outside the scope of this review.

## Unsupervised Methods

### Principal Component Analysis and Factor Analysis

Principal Component Analysis (PCA) attempts to explain variation in data using linear combinations of variables. It is a “dimension reduction” procedure, often used to reduce the number of variables to a smaller number of “components,” which account for most of the variation in the data. In contexts such as metabolomics, where hundreds of metabolites may be investigated for their relationship to some disease state, it can be difficult to conceptualize their role just because of their sheer number. PCA looks for underlying latent components or factors, which represent linear combinations of variables, but without predicting a dependent variable. The objective is to find linear combinations of variables that explain most of the variation in the data, in the process reducing the number of separate variables in the data (“reducing dimensionality”) (1, 32–34). These components or factors are expressed as “eigenvalues,” which in PCA are represented as linear combinations of the original variables, each with a coefficient or “eigenvector” that indicates the “direction” of that particular variable for each component. An important attribute is that the different PCs are uncorrelated or “orthogonal” (32–34), and this property means that they can be used in other statistical techniques such as discriminant analysis, where correlation between independent variables can be a problem (see orthogonal partial least squares discriminant analysis below in Orthogonal Partial-Least Squares Discriminant Analysis).

For each PC, each of the original independent variables is expressed in a linear equation with specific coefficients that represent the “weighting” of the variable in that component. The number of PCs, which can be large, is usually displayed in decreasing order of importance in explaining the variability in the data matrix, often shown graphically as a “Scree plot.” PCA is often performed using the correlation matrix for the data, in which case the data have to be standardized, i.e., each value subtracted from the mean for that variable and divided by the standard deviation (i.e., “z scores”). This prevents extreme differences in variance, e.g., due to different measurement scales, disproportionately affecting the analysis. While PCA is an exploratory method that does not make very many assumptions, the related method, Factor Analysis, has a formal statistical model, and assumptions such as multivariate normality (see below in Linear Discriminant Analysis) become important.

The interpretation of the PCs relies on the magnitude of the eigenvalues, and the contrasts between the eigenvectors for the variables relating to that eigenvalue. There is no specific criterion for how many PCs should be used; however, ideally, there should be a small number of PCs that explain most of the variation of the data. Loading plots, which represent the variance or magnitude of the variables within a PC, are often used to compare the different variables in the first two or three components. Because the interpretation of the PCs relies on the loadings, sometimes “rotations” are used to maximize the contrasts between them while maintaining the relationship between the variables in the components. Examples include “varimax” and “quartimax” rotations (1, 32–34).

Whether PCA is of any use in the analysis of multiple variables, depends on whether considering the different variables together, as a component, makes sense in the context of the research question, and also on what meaning can be attributed to the differences between the loadings. For example, if changes in free radicals are related to SNHL, does it make sense to reduce chemicals related to free radical generation to single components that combine the individual variables, or does this lose information? This problem of interpretability, which undermines many MVAs, will be addressed in a later section (see section Data pre-processing and imputation, overfitting and the problem of interpretability). PCA is often useful in a context where there are hundreds of variables, e.g., genomics, metabolomics, where it is useful to determine whether there is a change in the overall pattern of genes or metabolites [e.g., (35)].

### Cluster Analysis

Another MVA method that has not been used extensively in the context of auditory or vestibular neuroscience, is cluster analysis. Cluster analyses are a type of non-parametric statistical analysis that is used to explore the natural groupings of variables in a data set (1). Therefore, assumptions such as multivariate normality and equality of the variance-covariance matrices (see below in Linear Discriminant Analysis) are not required (1, 36). Different measurements of the distance between the variables, such as squared Euclidean or Mahalanobis distance, are used to relate them to one another, and specific algorithms (e.g., Ward Minimal Variance Linkage) are used to determine the clusters (36). As with PCA, the standardized data (i.e., z scores) are usually used in order to prevent bias introduced by differences in scales of measurement. The results are usually displayed using a “dendrogram.” Some cluster analysis algorithms, such as single linkage, are susceptible to producing long strings of clusters (“chaining”) (1, 36). Ward's Minimal Variance Linkage method, based on the objective of obtaining the smallest within-cluster sum of squares (the “minimal variance principle”), is often a good option (1, 36). The results of PCA and cluster analysis are often related, and it can be observed that many of the original independent variables that co-vary closely together in the dendrogram, also appear to have similar eigenvectors in the dominant PCs. In this way, PCA and cluster analysis provide similar information regarding which variables “work together,” but in different ways.

### K-Nearest Neighbors Algorithm

The K-Nearest Neighbors (“*k-*NN”) Algorithm can be used for regression or classification purposes. It is a non-parametric procedure in which either a category (in the case of classification) or a continuous variable value (in the case of regression) is estimated on the basis of its “nearest neighbours,” where “*k*” is usually a small positive integer (2, 37). The data are usually standardized (see above) before the analysis is performed. The main challenges of this method include determining the appropriate value for *k* (i.e., how many neighbors?) and how the distance between neighbors should be quantified [see (37) for a discussion]. It is possible to use the *k-*NN algorithm in unsupervised or supervised forms.

## Supervised Methods

### Regression

#### Multiple Linear Regression

Another statistical method that has been under-employed in auditory and vestibular neuroscience is multiple linear regression (MLR). MLR is a part of the general linear model (GLM), that is useful for determining whether one continuous variable can be predicted from a combination of other variables. Simple linear regression can be expanded to include more than one predictor variable to become MLR, which has the general form: *Y* = β_0_ + β_1_*X*_1_ + β_2_*X*_2_ +… β_p_*X*_*p*_ + ε, where *Y* = the continuous dependent variable; *X*_1_*, X*_2_*,…X*_*p*_ are independent variables; β_1_, β_2_… β_p_ are coefficients; β_0_ is the intercept and ε is the error term (2, 36–41).

Canonical correlation analysis is an extension of MLR in which multiple Y variables are related to multiple X variables (1).

Formal statistical hypothesis tests for MLR, like those for simple linear regression, make assumptions regarding the distribution of the data, which cannot always be fulfilled (see section Data pre-processing and imputation, overfitting and the problem of interpretability). These assumptions are the same as those for other methods that are part of the GLM, such as analysis of variance (ANOVA): that the residuals are normally distributed, with homogeneity of variance, and that they are independent of one another (e.g., not autocorrelated) (31, 36–43). Furthermore, the predictor variables should be numerical, although indicator variables can be used in order to include nominal variables (e.g., binary coding to represent male and female). The violation of the assumption of normality can sometimes be redressed using data transformation, which may also correct heterogeneity of variance, but other issues such as autocorrelation, are not easily dealt with and may require methods such as time series regression (31, 36–43).

Unlike simple linear regression, MLR is more complicated in terms of avoiding potential artifacts. Because the coefficient of determination, the *R*^2^, which indicates as a percentage or fraction, how much of the variation in the dependent variable is explained by the independent variables, will increase as more independent variables are incorporated into the regression model, an “adjusted *R*^2^” must be used in order to compensate for the number of variables included. The adjusted *R*^2^ = [R^2^-(k/n−1)][(n−1)(n–(k + 1))]. For k = 1 variables, the *R*^2^ and adjusted *R*^2^ are approximately equal.

There are various forms of MLR: forward regression, backward regression, stepwise regression and best subsets regression. In forward regression, predictors are added into the model one at a time (if α is set to 1.0, then all of them will be included, in ascending order of significance). In backward regression, predictors are taken out one at a time (if α is set to 0, all of them will be taken out, in descending order of significance). Backward regression tends to be preferred over forward regression because it allows examination of the interaction between variables (31, 36–43). In stepwise regression, the program stops at each step and checks whether the variables, either in the model or not, are the best combination for that step. The adjusted *R*^2^ will change as different variables are included in a model and an ANOVA can be done at each step to determine whether it has made a significant difference. Best subsets regression, however, computes all possible MLR models from which the researcher must choose the best, based on the adjusted *R*^2^ and various diagnostic information regarding the validity of the regression model (31, 36–43). One of the greatest problems in MLR is “over-fitting” and “multicollinearity” (31, 36–39, 44–46). If the regression variables are highly inter-correlated, multicollinearity occurs. This inflates the variance of the least square estimates and therefore the coefficients will be inaccurate, which can lead to the situation in which the ANOVA for the regression is significant without any single *t*-test for an individual variable being significant. In this case, one or more of the highly correlated variables needs to be removed from the regression model. One way of controlling for multicollinearity is using an index such as the Mallow's Cp index. The adjusted *R*^2^ should be high but the Mallow's Cp index [= (the sum of squares for the error at the current step/mean square error for the full regression)–(*n*−2p), where *n* = total number of observations and *p* = number of estimated coefficients], should be as small as possible. Ideally, it should be one more than the number of parameters in the current step. Other indices of multicollinearity include the variance inflation factor and tolerance (1/variance inflation factor) (36–39, 45, 46). Different software packages (e.g., SPSS and Minitab) offer different options. Autocorrelation in the data can be tested using the Durban-Watson statistic (36–39, 45, 46). Like most other multivariate statistical procedures, MLR is prone to artifacts and researchers need to be cautious when using it [see (44) for a rigorous discussion of this issue].

#### Random Forest Regression

Although modeling using regression trees has been used for over 25 years, its use in auditory and vestibular neuroscience has been very limited. In regression tree modeling, a flow-like series of questions is asked about each variable (“recursive partitioning”), subdividing a sample into groups that are as homogeneous as possible by minimizing the within-group variance, in order to determine a numerical response variable (47, 48). The predictor variables can be continuous variables also, or they can be categorical. By contrast with MLR, which makes assumptions about the distribution of the data, regression trees make no distributional assumptions. The data are sometimes split into training and test data sets (e.g., 70:30) and the mean square error between the model based on the training data and the test data, is calculated as a measure of the model's success. Variables are chosen to split the data based on the reduction in the mean square error achieved after a split (i.e., the information gained). Unlike MLR, interactions between different predictor variables are automatically incorporated into the regression tree model and variable selection is unnecessary because irrelevant predictors are excluded from the model. This makes complex, non-linear interactions between variables easier to accommodate than in linear regression modeling (47, 48). Breiman et al. (48) extended the concept of regression trees by exploiting the power of computers to simultaneously generate hundreds of trees (“bagging”), known as “random forests,” which were based on a random selection of a subset of data from the training set. The various regression tree solutions are averaged in order to predict the target variable with the smallest mean square error (47–52). An alternative form of cross-validation of the random forest model, which does not require splitting the data set and therefore is particularly useful in the context of small sample sizes, is the leave-one-out (“LOO”) procedure. Here, each subject is removed from the sample, in turn, and the model based on the remaining data is used to predict for that subject; then, another subject is removed, and the procedure repeated, until the entire data set has been cross-validated (47–51).

Gradient boosted decision trees (GBDTs) are an alternative to the random forest procedure in which learning algorithms are combined (“boosting”) so that each decision tree tries to minimize the error of the previous tree (37, 52).

### Classification

#### Logistic Regression

Logistic regression is similar to linear regression but applied to the prediction of a binary outcome (37, 39). Rather than fitting a linear function to the prediction of a continuous dependent variable, logistic regression employs the logistic function, logistic(η) = 1/(1 + *exp*(*-*η)), to generate an outcome between 0 and 1. The logistic function is then incorporated into the probability function, *P*(*y*^(*i*)^ = 1) = 1/(1 + *exp* (–(β_0_ + β_1_ $x_{1}^{\left(i\right)}$ + …. β_*p*_ $x_{p}^{\left(i\right)}$))), where *P* is a probability, *x* are predictor variables, the β'*s* represent coefficients and β_0_ is the intercept (37). The output of this function is then a probability of classification to one of two groups, although logistic regression can also be extended to multinomial regression (39).

#### Linear Discriminant Analysis

Linear discriminant analysis is a statistical method that is often used to predict the membership of two or more groups from a linear combination of independent variables (1, 2). A linear discriminant function (LDF) has the general form: Z = a_1_X_1_ + a_2_X_2_ +…a_p_X_p_, where Z refers to the group, X, X_2_,…X_p_ are independent variables, and a_1_, a_2_,…a_p_ are coefficients (1). Linear discriminant analysis is similar in aim, but different in approach, to logistic regression, in which the dependent variable is binary (0/1) and consists of positive (a “success”) and negative responses (a “failure”) only (1). An example in the context of auditory neuroscience might be the prediction of SNHL by a linear combination of neurochemical variables in the peripheral or central auditory systems [e.g., (35)]. The statistical significance of the LDF can be assessed using statistics such as Wilk's λ and its success in separating the groups can be evaluated using cross-validation (e.g., a LOO procedure), in which the linear equation is used to classify the data, one observation at a time, without knowledge of the actual group membership. It is possible to use a stepwise linear discriminant analysis. However, some authors [e.g., (1, 45, 46)] suggest that stepwise methods can result in suppressor effects and an increase in type II error. Linear discriminant analysis is readily available in programs such as SPSS and Minitab. It is part of the GLM, and therefore makes similar assumptions to MLR, but other forms of discriminant analysis, which do not make all of these assumptions, include quadratic discriminant analysis, where the data are assumed to be normally distributed but the variance-covariance matrices need not be identical. Orthogonal partial least squares discriminant analysis is another type of discriminant analysis in which the discriminant function consists of PCs from a PCA (see below in Orthogonal Partial-Least Squares Discriminant Analysis).

As mentioned, MVA methods that are part of the GLM, such as linear discriminant analysis, do make assumptions. The first is that, for formal tests of statistical significance to be valid, the data within groups should have a multivariate normal distribution (1). Unlike univariate statistical analyses such as ANOVA, linear discriminant analysis is quite sensitive to the violation of the assumption of multivariate normality (1, 2, 36, 53). It is difficult to test for multivariate normality, because most programs such as SPSS do not offer such an assumption test (1). Because univariate normality, i.e., the normality of the individual variables, is necessary but not sufficient for multivariate normality, it is possible for each individual variable to be normally distributed without the multivariate distribution being normally distributed. Stevens (2) points out that because a multivariate normal distribution entails that all subsets of variables have normal distributions, one way to assess multivariate normality is to determine whether all pairs of variables are bivariate normal. Box's test for the homogeneity of the covariance matrices (see below) is sensitive to violation of multivariate normality; therefore, in order to obtain results from that test that are valid, whether the assumption of multivariate normality is fulfilled, must be of concern (2). However, there is a multivariate formulation of the central limit theorem and sample sizes of 10–20 per group appear to be sufficient to afford protection against the consequences of violating multivariate normality (2, 45, 46). It should be noted that linear discriminant analysis may still discriminate between groups even if the assumption of multivariate normality does not hold. On the other hand, multivariate normality does not necessarily mean that it will effectively discriminate between the groups.

A second assumption of linear discriminant analysis, but not quadratic discriminant analysis, is that the population covariance matrices are equal for all groups, usually tested using Box's *M*-test (1, 36). If this assumption is violated, a quadratic discriminant analysis, can be used instead. In a review of several Monte Carlo studies, Stevens (2) concluded that, provided that the sample sizes are equal, even moderate heterogeneity of the covariances does not substantially affect type I error. Unequal sample sizes, on the other hand, are potentially very problematic if the covariances are unequal (2). While Box's *M*-test is often used, its null hypothesis may be rejected only because the multivariate normality assumption is violated (2). Therefore, it is important to determine whether this is the reason for a significant Box's *M*-test. Box's *M*-test is also very sensitive to departure from homogeneity of the covariances (45, 46). Both Stevens (2), Field (45), and Field et al. (46) suggest that even if Box's *M*-test is significant, the type I error rate will be only slightly affected provided that there are equal sample sizes, although the power may be somewhat reduced.

One of the common problems in many MVAs is the sample size for each variable, n, relative to the number of variables, *p*. While unequal sample sizes can be problematic, as described above, when *p* is greater than *n*, statistical analyses such as linear discriminant analysis can become invalid. Stevens (2), Field (45), and Field et al. (46) suggest that, unless the n is large, *p* ≤ 10. Monte Carlo studies have shown that if the sample size is not large compared to the number of variables, the standardized discriminant function coefficients and correlations obtained in a linear discriminant analysis, are unstable (2). By “large,” Stevens (2) suggests a ratio of *n* (total sample size):*p* (number of variables) of 20:1. He further cautions that a small *n:p* ratio (i.e., ≤5) can be problematic for stepwise linear discriminant analysis in particular, because the significance tests are used to determine which variables are included in the solution (2).

These methods, and others related to them such as orthogonal partial least squares discriminant analysis, should be applicable to many situations in auditory and vestibular neuroscience in which multiple variables interact to determine a categorical dependent variable, e.g., SNHL, tinnitus, Meniere's Disease, vestibular neuritis, and benign paroxysmal positional vertigo, provided that the sample sizes are sufficient and the cross-validations demonstrate the predictive accuracy of the LDFs. Given that Box's *M*-test of the equality of the covariance matrices assumes multivariate normality, one way to proceed is to determine whether all pairs of variables appear to be bivariate normal. If so, Box's *M*-test can be used as a guide to whether the assumption of the equality of the covariance matrices is fulfilled. However, the cross-validation procedure can be used as the ultimate arbiter of the effectiveness of the LDF (31).

#### Random Forest Classification

The random forest method that is used for regression, can also be used for classification purposes, in which case the solution is based on the number of “votes” from the different trees for a particular category (48, 49). The effect of variable removal on the mean decrease in accuracy, the “out of bag” (“OOB”) error, and the overall classification matrix error (“confusion matrix error”), are used to evaluate the success of the classification. The OOB error is the error based on the observations that were excluded from the subset of the training data (the “bag”) used to generate the decision tree (47, 48). Unlike linear discriminant analysis, random forest classification makes no distributional assumptions and therefore can be applied to situations in which the sample sizes are small relative to the number of variables (47, 48). Random forest classification, along with support vector machines, can be carried out using specific packages in the statistics program, R (47, 54–57). For those who do not wish to use code in R, there is a data mining graphics user interface available, called “Rattle,” which is menu-driven and easy to use (55).

#### Support Vector Machines

Support vector machines are an alternative method for classification, which employ “support vectors,” observations that form the spatial boundary between different classes (47–49, 54). These support vectors are then used to determine a hyperplane that defines the boundary between the classes (46–54). Support vector machines can employ a variety of functions, such as radial kernel and Laplace functions, to remap the data and generate new variables that can separate the different categories (47–54). The data are usually split into training and test data sets (e.g., 70:30) and the difference between the model based on the support vectors in the training data set, and the test data set, is calculated as a measure of the model's success. As with linear discriminant analysis, classification error matrices can be used to evaluate the success of the classification, as well as receiver operating characteristic (“ROC”) curves, that quantify the relationship between the true positive rate of classification (“sensitivity”) and the false positive rate of classification (“1—the specificity”) (47).

One of the major advantages of support vector machines is that they do not make distributional assumptions like linear discriminant analysis, other than that the data are independent and identically distributed. Wilson (54) suggests that for this reason, even small sample sizes can provide accurate estimates of prediction error when there are a large number of variables.

#### Bayesian Classifiers

Bayesian classification methods are based on Bayes' Theorem, which relates a posterior probability of an event to a prior probability: P(H/X) = P(X/H)(P(H)/P(X), where X represents the data and H represents the hypothesis; P(H/X) = the probability of H given X (the posterior probability), P(X/H) = the probability of X given H, P(H) = the probability of H (the prior probability), and P(X) = the probability of X, which cannot = zero. P(H/X) and P(X/H) are known as “conditional probabilities” and P(X) and P(H) as “marginal probabilities” (58–61). In simple terms, Bayes' Theorem relates the degree of belief in an hypothesis before accounting for the data, to that after accounting for the data, so that the probability of the hypothesis being true given the data, equals (the probability of obtaining the data given that the hypothesis is true, multiplied by the probability that the hypothesis is true), divided by the probability of obtaining the data (58–61). The calculation of the conditional and marginal probabilities can be used to generate a Bayesian Network, which can be displayed in graphical form such as directed acyclic graphs (61).

#### Orthogonal Partial-Least Squares Discriminant Analysis

Also known as orthogonal projection to latent structures discriminant analysis, orthogonal partial-least squares discriminant analysis (OPLS-DA) is a method of discriminant analysis that cleverly combines PCA with discriminant analysis and partial least squares regression, in order to classify subjects (62). Therefore, it can be seen as an alternative to methods such as linear discriminant analysis, support vector machines and random forest classification. OPLS-DA is an ideal method to use in the search for biomarkers of SNHL, tinnitus or vestibular disorders, for e.g., using metabolomic data from animals or humans with those conditions. In partial least squares regression, which is an extension of MLR, factors are extracted from the *Y*′*XX*′*Y* matrix in order to generate prediction functions, only in the case of partial least squares discriminant analysis, the dependent variable is categorical. One major advantage of partial least squares discriminant analysis is that it is minimally restrictive because it allows for fewer observations than variables (i.e., less *n* than *p*), a problem that is significant for linear discriminant analysis (62). As with PCA and cluster analysis, the data would normally be standardized to z scores before proceeding.

In OPLS-DA, the X variables are latent variables that maximize the separation between the groups, ranked according to how much variation in Y that they explain. OPLS-DA separates the systematic variation in X into 2 parts: (1) that which is linearly related to Y; and (2) that which is unrelated or “orthogonal” to Y (62). The OPLS method uses a modification of the non-linear iterative partial least squares algorithm (62). An orthogonal signal correction procedure, developed by Trygg and Wold (62), employs an iterative process to find orthogonal components in the X matrix. For this it depends on a starting vector, which can use PCs from a PCA. The main problem with discriminant analysis is over-fitting, particularly where the *p* > the *n*, so that the model works well on the training data but not on new data, and where there is multicollinearity. However, this possibility can be addressed using permutation testing (63). In permutation testing, variables are assigned randomly to the different samples, and new models are generated many times, e.g., 2,000. A null distribution of classifications is created, which is expected to be non-significant. The results obtained from the original data should be outside the 95 or 99% confidence intervals for the null distribution, in order to be statistically significant, i.e., not part of the null distribution (63).

Biplots can be used to show some of the results of an OPLS-DA, where the x axis is labeled “t[1],” which are the X scores predictive of Y, and the y axis is labeled “to[2],” which are the X scores that are not predictive of Y. Therefore, the x axis represents the between group variation and the y axis represents the within group variation. OPLS-DA calculates various indices of the success of the model. *R*^2^*X* (cum) is the sum of the predictive and orthogonal variation in X that is explained by the model, which can be split into the predictive and orthogonal components. *R*^2^*Y* (cum) is the total sum of variation in Y explained by the model. *Q*^2^ is the effectiveness of the prediction, based on the OPLS-DA equation, using cross-validation, e.g., using a LOO procedure, where 0.9 would be excellent.

S plots are often used to help interpret the OPLS-DA results. In the S plot, the x axis “p[1]” is the magnitude of each variable in the x axis. The y axis “p(corr)[1]” is the reliability (obtained with confidence intervals using jack-knifing and cross-validation). Values close to zero on both axes are close to noise, i.e., they have almost zero magnitude and reliability. OPLS-DA can be carried out in R, or using the programs Metaboanalyst and Metscape 3.1 (35, 64).

Pathway impact analyses can also be carried out on the OPLS-DA. Using the prior knowledge of pathways, these methods look for over-representation of specific pathways in the data. They calculate the sum of the importance measures of the matched metabolites normalized by the sum of the importance measures of all metabolites in each pathway. Over-representation analysis, quantitative enrichment analysis, and single sample profiling are three different types of pathway analysis that can be used in the program Cytoscape 3.30 (35, 64, 65).

## Data Pre-Processing and Imputation, Overfitting, and the Problem of Interpretability

When using MVA and data mining methods to analyse data, some pre-processing of the data is often necessary. In the case of MVA, methods that are part of the General Linear Model (GLM), such as multiple linear regression, multivariate multiple linear regression, linear discriminant analysis, structural equation modeling, and canonical correlation analysis, require that the assumption of multivariate normality be met (see section Linear Discriminant Analysis). Therefore, data need to be checked to determine whether they are normally distributed or even whether they have a multivariate normal distribution (see Linear Discriminant Analysis). Normality (Q–Q) plots and plots of residuals vs. fitted values usually need to be obtained and formal assumption tests conducted, such as the Anderson-Darling, Shapiro-Wilk or Kolmogorov-Smirnov tests for univariate normality, and Bartlett's or Levene's tests for homogeneity of variance. If these tests are statistically significant (i.e., *P* ≤ 0.05), a decision may be made to transform variables in order to achieve fulfillment of the normality and homogeneity of variance assumptions; however, great care needs to be taken in transforming non-linear dependent variables into linear ones, as in Scatchard plots, because of the way that it can distort the error around the line of best fit (66). For receptor binding data, non-linear regression is now considered preferable to linear regression following transformation (66). In the case of other methods such as PCA, OPLS-DA and cluster analysis, pre-processing may involve standardizing the data (see Principal Component Analysis and Factor Analysis), in order to ensure that differences in measurement scales do not bias the analysis.

Even for univariate statistical analyses, many statistical programs delete experimental subjects if they have missing data for procedures such as repeated measures ANOVAs (43, 45). Many animal studies in auditory and vestibular neuroscience already have small and unequal sample sizes; therefore, simply deleting data in the case of missing values will result in lower statistical power and may bias the results (67). For alternatives to repeated measures ANOVAs such as linear mixed model analysis, “imputation” procedures are employed in order to estimate the missing values (“Missing Values Analysis or MVA”) (43, 67, 68). A maximum likelihood (ML) and expectation-maximization (EM) approach (a combination of imputation and ML) can be used (68). However, only some programs (e.g., SPSS) offer the EM algorithm and for the ML and EM methods to be used, the missing data must be “missing at random” (MAR, i.e., the probability that an observation is missing must not depend on the unobserved missing value but may depend on the group to which it would have belonged) or “missing completely at random” (MCAR, i.e., the probability that an observation is missing must not depend on the observed or missing values) (67, 68). In other words, there can be no bias to the way that data are missing, a condition that is sometimes difficult to satisfy. The K-NN algorithm discussed in K-Nearest Neighbors Algorithm can be used for imputation and there is a variety of multivariate imputation procedures [see (69) for a review].

“Overfitting” is an enormous problem in MVA and data mining methods which involve regression modeling. Overfitting occurs when a model for prediction is based so closely on a particular data set that it has little predictive value for other, similar data sets, often a result of including too many parameters in the model (44). As a result, a regression model based on a training data set may have no predictive value for the test data set. Although the problem is well-recognized in MLR (44), Breiman et al. (48) have suggested that random forest methods do not overfit, a view that has been challenged (70). Solutions to overfitting include collecting more data so that there is a larger n for each predictor variable, *p*, combining predictors in order to reduce correlation between them and the use of “shrinkage and penalization” procedures (44). The adjusted *R*^2^ in MLR is one type of shrinkage estimator because it takes into account the number of predictor variables. “Lasso” regression (“least absolute shrinkage and selection operator”) is a method that generates a linear regression model with greater “sparsity” [see (37) for a review].

One of the advantages of univariate statistical methods is that they are relatively easy to understand and this is partly why they are so popular. Researchers turn to multivariate statistical and data mining (or machine learning) methods because they have to deal with many variables, sometimes hundreds or thousands, but in the process of using such procedures, they sacrifice simplicity and interpretability. Molnar (37) has written extensively about the problem of “interpretability” with MVA and data mining methods, which involve complex modeling. Even if they provide good predictive value, they may be difficult to understand. Simpler models, by definition, such as shorter decision trees, are more easily interpreted than longer ones. Molnar (37) has suggested that “model agnostic interpretation methods” be used for machine learning in preference to “model-specific ones.” These are methods that can be applied to any machine learning model, are not restricted to a certain form of explanation (e.g., a linear formula vs. a graphic representation) and should have flexibility in the way that the explanation is represented. Examples include partial dependence plots (PDP), feature importance and Shapley values [see (37) for a review].

## Examples of Applications of Multivariate Statistical and Data Mining Methods to the Analysis of Otological Data

### Principal Component Analysis and Cluster Analysis

**Figure 3:**
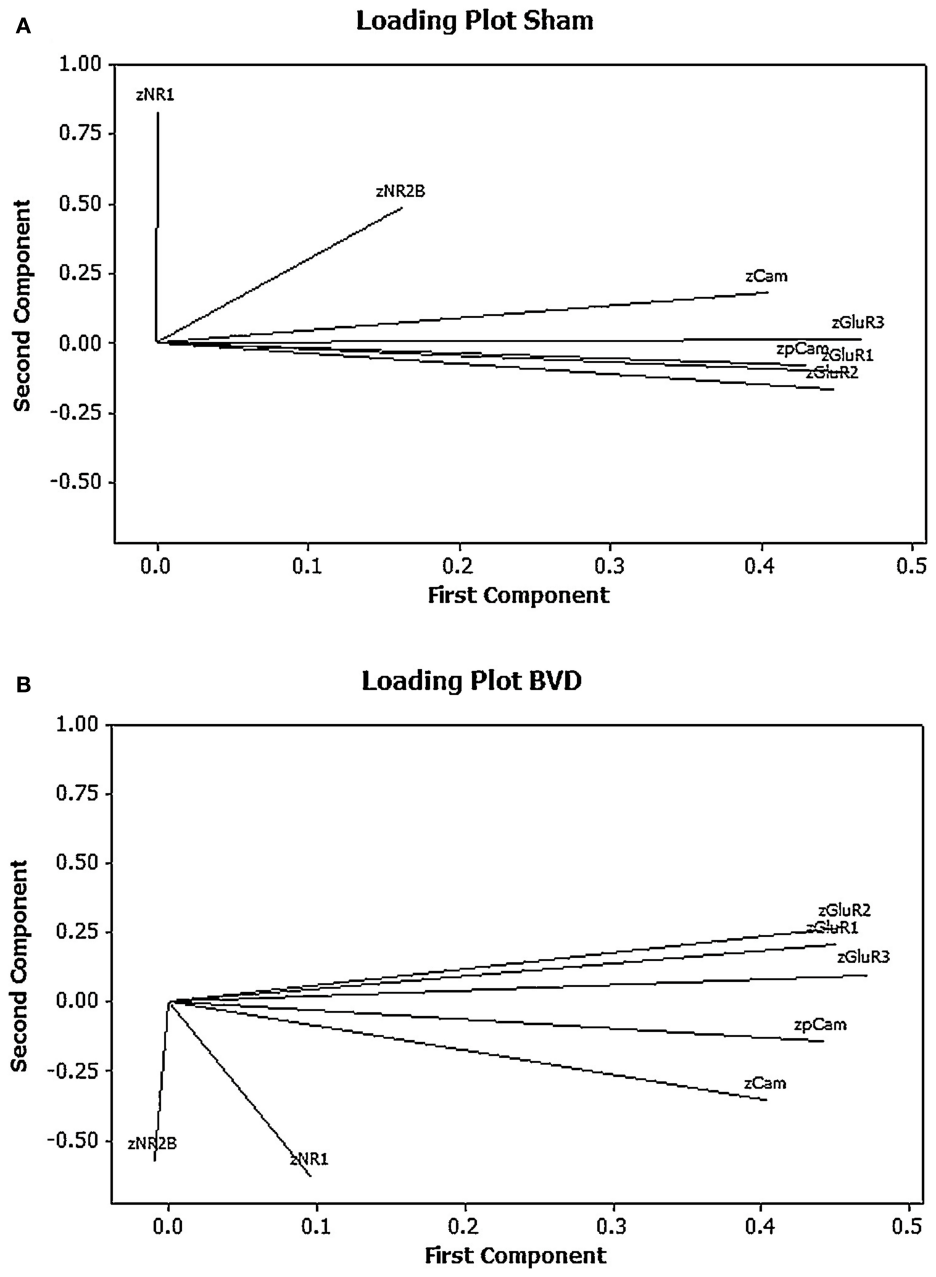
Loading plots for the first 2 principal components for the mean normalized density of expression of NR1, NR2B, GluR1, GluR2, GluR3, CaMKII, and pCaMKII in the CA1, CA2/3, and dentate gyrus (DG) regions of the hippocampus at 6 months following sham **(A)** or BVD **(B)** surgery. Note the inverted pattern of loadings for the BVD group compared to the sham group. From Smith and Zheng (6) with permission.

One of our research interests has been the role of neurochemical changes in the hippocampus in the cognitive deficits that occur following peripheral bilateral vestibular damage. In this process we have used western blotting to analyse the expression of various glutamate receptor subtypes in the rat hippocampus, given the importance of glutamate receptors to memory processes such as long-term potentiation [see (71) for a review]. Due to the fact that we quantified 5 different glutamate receptor subunit subtypes (GluR1, GluR2, GluR3, NR1, and NR2) and 2 forms of calmodulin kinase II (CaMII and phosphorylated CaMII), related to glutamate receptor activation, we decided to use PCA and cluster analysis to analyse the results, particularly so that we could understand the co-variation and interactions of any changes in the expression of the proteins. Using univariate statistical analysis, there were no significant differences in the expression of any individual protein between the bilateral vestibular deafferentation (BVD) group and the sham controls (15); however, PCA suggested that when the 1st and 2nd components were plotted against one another using a loading plot, the relationship between the expression of the different proteins had changed [see Figure 3; (6)]. Although the meaning of this shift is not easy to interpret—one of the perennial problems of PCA—this MVA revealed a change in the pattern of interaction between the different proteins which the univariate analysis could not. Note that all of the data were transformed to z scores.

**Figure 4:**
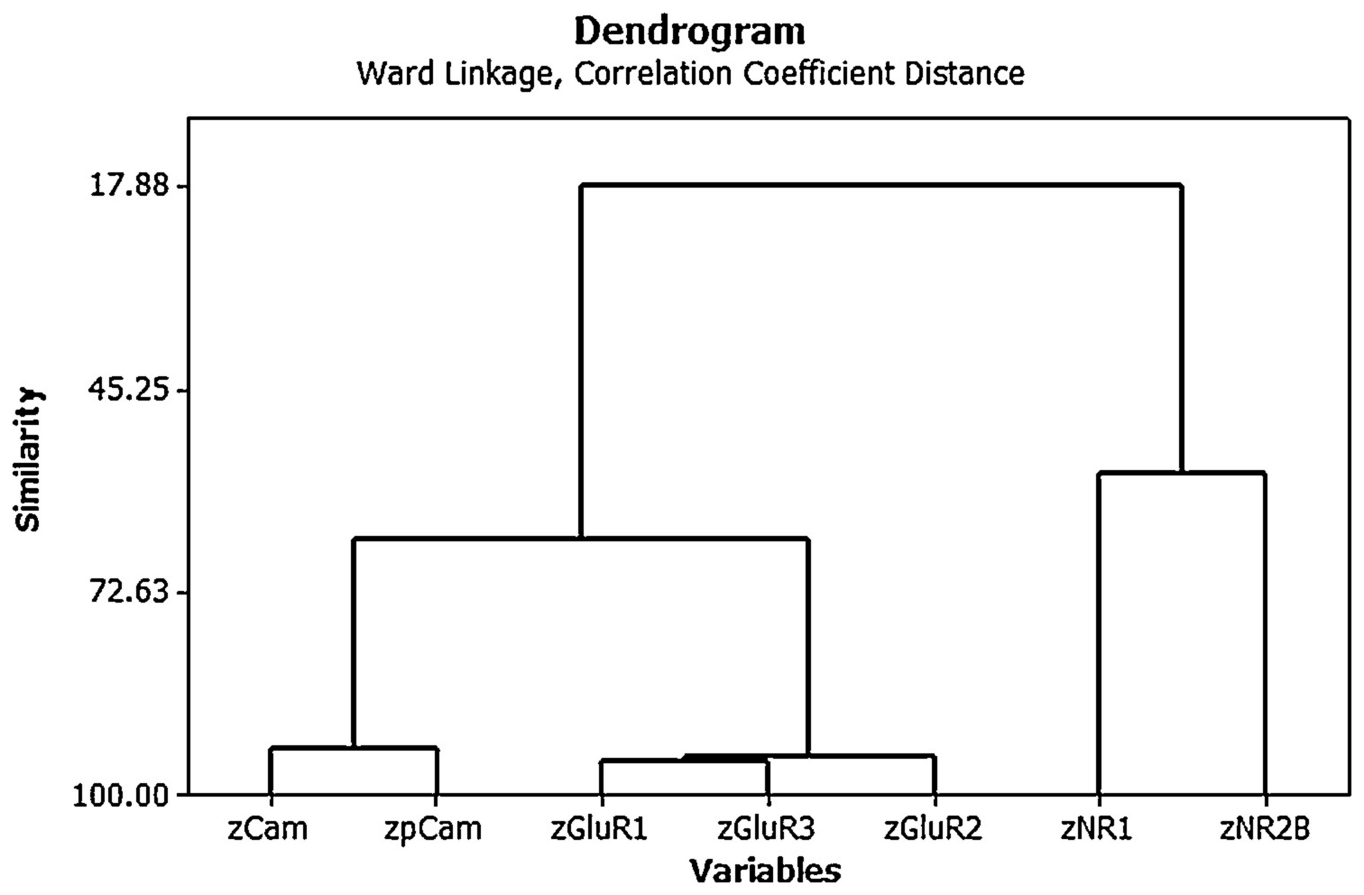
Ward's minimal variance cluster analysis on the correlation coefficient distance for the mean normalized density of expression of NR1, NR2B, GluR1, GluR2, GluR3, CaMKII, and pCaMKII in the CA1, CA2/3, and dentate gyrus (DG) regions of the hippocampus at 6 months following BVD or sham surgery (i.e., both groups shown together). The *y* axis shows degrees of similarity, where 100.00 is the highest similarity and 17.88 is the lowest. Note that the NMDA receptor subunits vary together, as do the AMPA receptor subunits, as well as the CaMKII isoforms. From Smith and Zheng (6) with permission.

Cluster analysis of the individual protein variables showed that they co-varied in a predictable way [see Figure 4; (15)]. Note again the use of z scores and the fact that the AMPA (GluR1, 2, and 3) and NMDA (NR1 and 2) receptor subunits tended to co-vary closely with one another.

### Multiple Linear Regression

Another area of interest for us has been the L-arginine cycle and its role in producing nitric oxide synthase, polyamines and glutamate in the brainstem vestibular nucleus and cerebellum (Figure 1). This complex pathway is involved in brain aging in the central vestibular system and has been the target for drug treatments aimed at interfering with neurodegenerative diseases such as Alzheimer's Disease (3, 4). Because these neurochemicals interact in a network, it is important to understand how each part of the system affects the other parts. We have used MLR in an attempt to predict different neurochemicals in this pathway from one another, with adjusted *R*^2^ values ranging from 0.50 (ornithine) to 0.95 (citrulline) (51). The best predictions were for citrulline (0.95), spermine (0.93) and arginine (0.92) (see Table 2). Assumptions were tested using normal Q–Q plots and residuals vs. fitted values plots, and were fulfilled. In this study, MLR was compared directly with random forest regression on the same data set (51) (see below).

**Table 2:** Results of the multiple linear regression analysis of the data from Smith et al. (51), showing the adjusted *R*^2^-values, the residual standard errors (RSEs) and the significant input variables.

	**GABA**	**put**	**spd**	**spm**	**arg**	**glut**	**agm**	**orn**	**cit**
*R^2^*	0.811	0.675	0.861	0.938	0.936	0.698	0.796	0.623	0.958
MSE	846.57	0.23	203.29	34.89	225.48	50064.54	0.12	189.54	119.52
Significant predictor variables	cit^***^	ag^***^	spm^***^	spd^***^	cit^***^	GABA^***^	put^***^	age^***^	arg^***^
	glut^***^	orn^*^	age^***^	cit^***^	orn^*^	spm^***^	cit^***^	cit^***^	GABA^**^
	spd^*^		orn^**^	glut^***^		orn^**^	age^***^	glut^**^	age^**^
			glut^*^					spd^**^	spm^**^
								enrich^*^	orn^**^

*** *P ≤ 0.0001*,

** *P ≤ 0.001*,

* *P ≤ 0.05*.

*From Smith et al. (51) with permission*.

### Random Forest Regression

**Figure 5:**
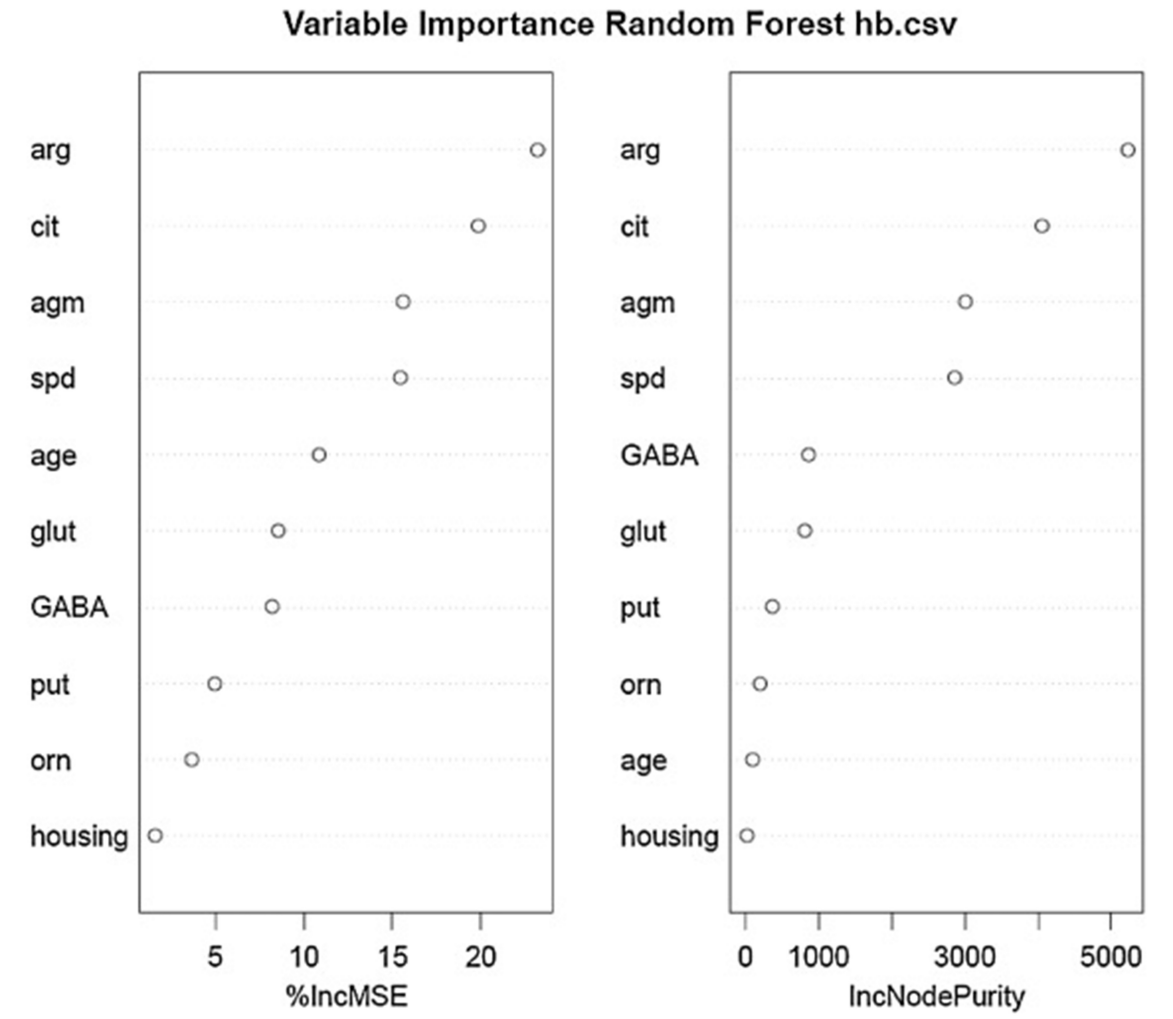
Variables in order of importance for the random forest regression analysis for spermine, which had the highest proportion of variance explained (0.94). From Smith et al. (51) with permission.

**Figure 6:**
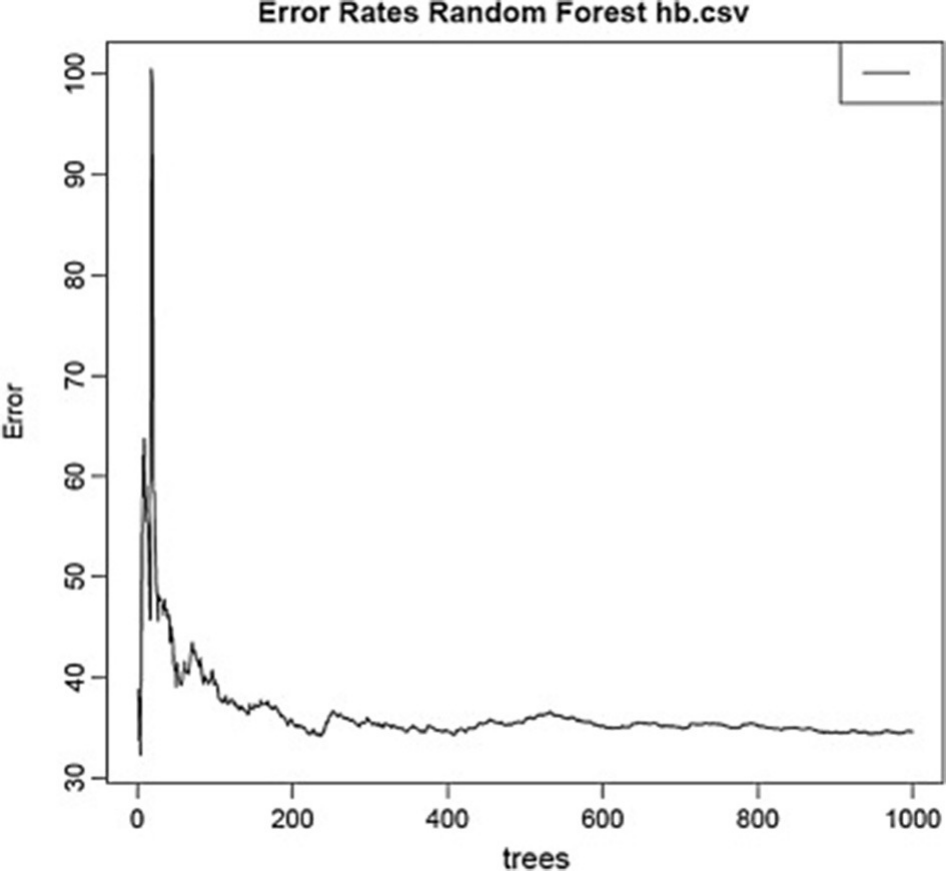
Decrease in error as a function of the number of trees for the random forest regression model for spermine, which had the highest proportion of variance explained (0.94). From Smith et al. (51) with permission.

Using the same data set, we found that random forest regression was also successful in predicting the neurochemical concentrations in the L-arginine pathway, with the best values for the proportion of variance explained, 0.94 (spermine), 0.92 (arginine), and 0.90 (citrulline) [see Table 3; (51)]. However, for this data set, random forest regression was somewhat less successful than MLR in predicting some of the variables (e.g., 0.27 for ornithine; see Table 3). Variable importance plots (VIPs) were obtained and Figure 5 shows the variables in order of importance for the prediction of spermine, where arginine and citrulline were clearly the most important variables (51). Figure 6 shows the degree of error in the prediction of spermine as a function of the number of trees generated. It can be seen that the error decreases rapidly after the first 150–200 trees (51).

**Table 3:** Results of the random forest regression models of the data from Smith et al. (51), showing the proportion of variance explained values, the residual standard errors (RSEs) and the input variables chosen using the stepwise process.

	**GABA**	**put**	**spd**	**spm**	**arg**	**glut**	**agm**	**orn**	**cit**
*R^2^*	0.939	0.868	0.947	0.989	0.986	0.914	0.910	0.861	0.983
MSE	271.63	0.09	77.38	6.14	47.96	14,285.84	0.05	69.75	48.63
Most important predictor variables	cit	agm	spm	arg	cit	spm	arg	age	arg
	glut	arg	arg	cit	spm	GABA	put	glut	spm
	arg		agm	agm		cit	spm	cit	agm
			cit					GABA	GABA
								arg	spd

*From Smith et al. (51) with permission*.

### Linear Discriminant Analysis

We have also used linear discriminant analysis to predict the age of animals based on the concentrations of neurochemicals in the L-arginine pathway (Figure 1). This research is directly applicable to the identification of biomarkers that might be used to predict pathological changes that occur in brain aging in vestibular areas of the brain. In Liu et al. (3), we identified an LDF that could predict whether rats were young (4 months old) or aged (24 months old). The LDF based on putrescine, spermidine, spermine, citrulline, glutamate and GABA in the vestibular nucleus (note the z transformation), could predict age with 100% accuracy using cross-validation (*P* = 0.000, Wilks'λ). Using the cerebellum, age could be predicted with 93% accuracy (*P* = 0.000, Wilks'λ), using only spermine and spermidine. Similar results were reported by Liu et al. (4), who found 90% accuracy in classifying animals to the aged group based on neurochemicals in the vestibular nucleus and 80% accuracy in classifying them based on neurochemicals in the cerebellum. We have also applied linear discriminant analysis to the prediction of whether rats have had a BVD or a sham procedure based on a combination of their behavioral symptoms, such as unsupported rearing, locomotor activity in the inner vs. outer zones of the open field maze and performance in the spatial alternation in a T maze task, and found that whether the animals had received a BVD could be predicted with 100% accuracy (*P* = 0.000, Wilks'λ) (5). These kinds of methods may be applicable to the differential diagnosis of vestibular and auditory disorders.

### Random Forest Classification

**Figure 7:**
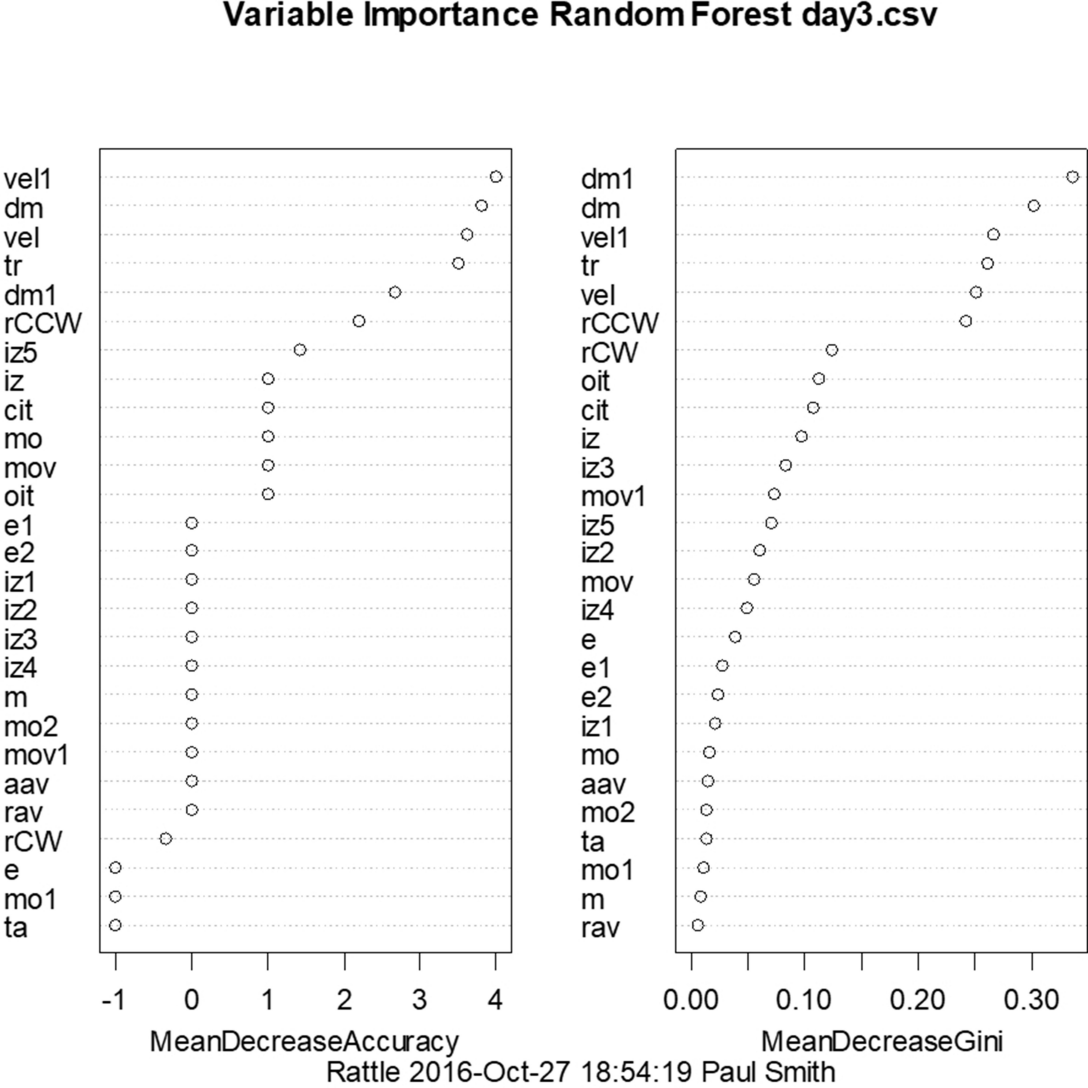
Order of variable importance for the random forest classification for day 3 following BVD, showing the decrease in the accuracy of the prediction as the variables are removed. The mean decrease in Gini coefficient is an indication of the extent to which each variable contributes to the homogeneity of the nodes and leaves in the random forest. dm, distance moved nose-point; dm1, distance moved center-point; e, contracted; e1, elongation normal; e2, elongation stretched; iz, in zone outer zone, nose-point; iz1, in zone inner zone, nose-point; iz2, in zone mid zone, nose-point; iz3, in zone outer zone center-point; iz4, in zone inner zone center-point; iz5, in zone mid zone center-point; m, meander center-point; cit, center inter-transition center-point; mo, mobility immobile; mo1, mobility mobile; mo2, mobility highly mobile; mov, movement center-point/moving; mov1, movement center-point/not moving; rCCW, rotation counter-clockwise center-nose; rCW, rotation clockwise center-nose; ta, turn angle center-point; vel, velocity nose-point; vel1, velocity center-point; aav, absolute angular velocity center-point; oit, outer inter-transition center-point; rav, relative angular velocity; tr, total rotations. From Aitken et al. (72) with permission.

With a similar aim to the use of linear discriminant analysis to predict whether animals have BVD on the basis of their behavioral symptoms, we have also employed random forest classification using a range of symptoms measured using the Ethovision tracking system (72). For days 3 and 23 post-BVD, we found that random forest classification could predict which rats had received BVD and which were sham animals with 100% accuracy. Figure 7 shows the variables in order of importance for day 3 and indicates that the most important variables were the animals' locomotor velocity (hyperactivity is a common symptom of BVD in rats), distance moved and rotation (72).

Ahmadi et al. (26, 27) have recently used logistic regression and random forest classification, as well as artificial neural networks, to support differential diagnosis of peripheral and central vestibular disorders in humans. In general, they observed that machine learning methods outperformed univariate scores. Karmali et al. (28) also used logistic regression to predict the probability of falling based on age and thresholds for the perception of 0.2 Hz roll head tilt.

### OPLS-DA

**Figure 8:**
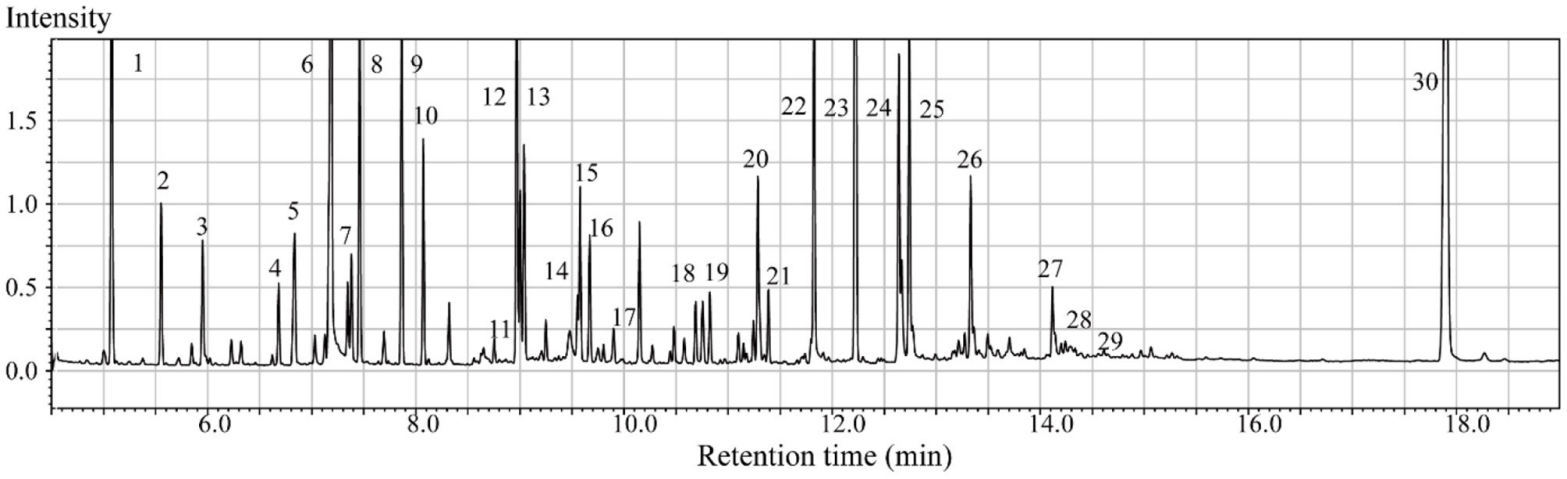
Typical GC/MS chromatograms of extracts from brain tissue of a sham animal. The compounds were identified as: 1. Lactic Acid, 2. Alanine, 3. Oxalic acid, 4. Valine, 5. Urea, 6. Phosphoric acid, 7. Proline, 8. Glycine, 9. Serine, 10. Threonine, 11. Malic acid, 12. Aspartic acid, 13. γ-Aminobutanoic acid, 14. Creatinine, 15. Glutamic acid, 16. Phenylalanine, 17. N-Acetylaspartic acid, 18. Hypoxanthine, 19. Citric acid, 20. Lysine, 21. Tyrosine, 22. Palmitic acid, 23. Myo-Inositol, 24. Oleic acid, 25. Stearic acid, 26. Arachidonic acid, 27. Docosahexaenoic acid, 28. Inosine, 29. Glycerol monostearate, 30. Cholesterol. From He et al. (35) with permission.

**Figure 9:**
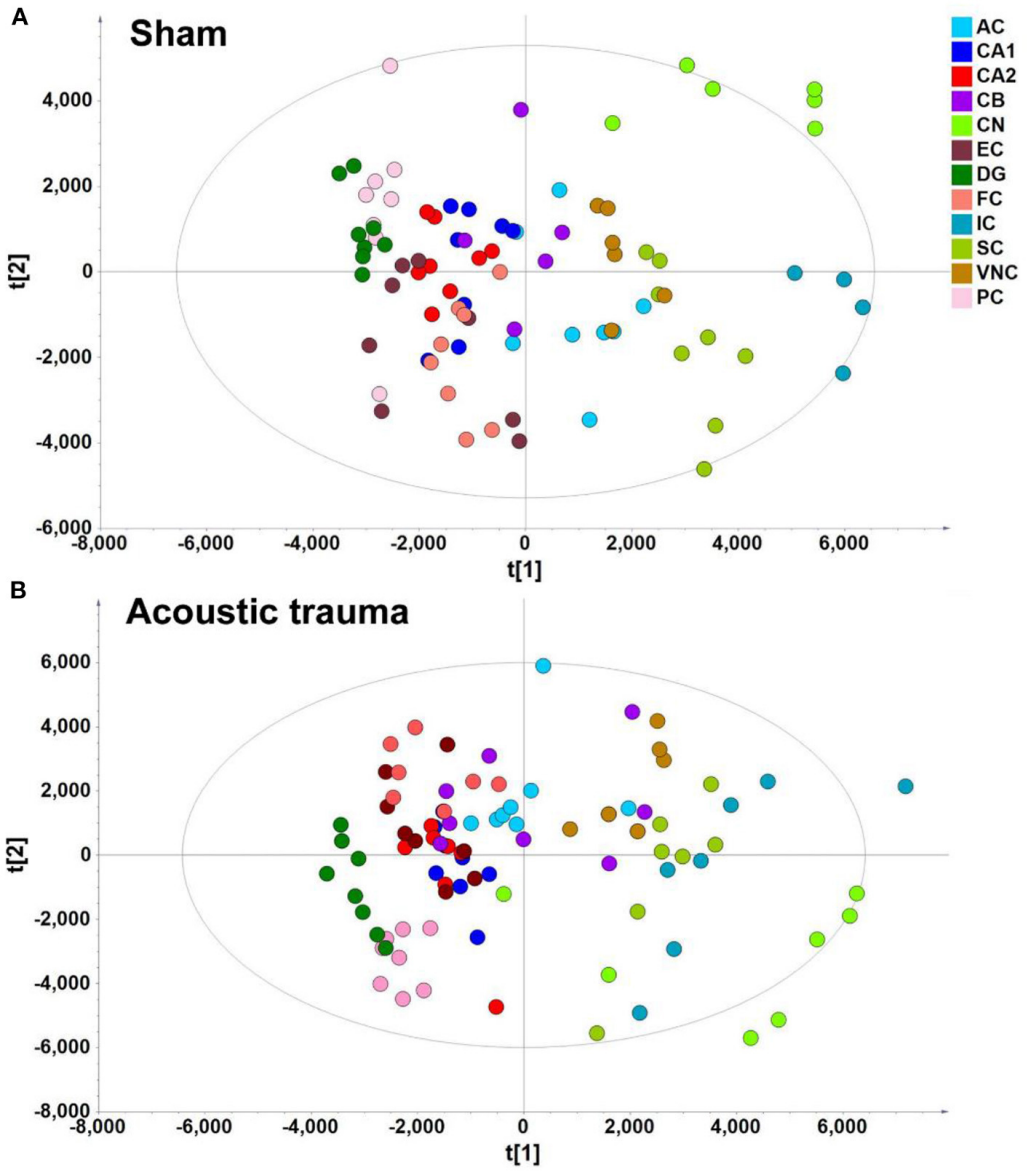
PLS-DA score plot of different brain regions from sham **(A)** and acoustic trauma **(B)** groups. **(A)** sham [PC1: *R2X* = 0.344, *R2Y* = 0.0842, *Q*^2^ =0.0726; PC2: *R2X* = 0.249, *R2Y* = 0.0525, *Q*^2^ = 0.0373; All 6 PCs: *R2X* (cum) = 0.84, *R2Y* (cum) = 0.381, *Q*^2^ (cum) = 0.286]; **(B)** acoustic trauma [PC1: *R2X* = 0.336, *R2Y* = 0.0759, *Q*^2^ = 0.0683; PC2: *R2X* = 0.307, *R2Y* = 0.0513, *Q*^2^ = 0.0407; All 6 PCs: *R2X* (cum) = 0.875, *R2Y* (cum) = 0. 384, *Q*^2^ (cum) = 0.325]. AC, auditory cortex; CB, cerebellum; IC, inferior colliculus; CN, cochlear nucleus; VNC, vestibular nucleus complex; SC, superior colliculus; CA1 and CA2 of the hippocampus; DG, dentate gyrus; FC, frontal cortex; PC, perirhinal cortex; EC, entorhinal cortex. From He et al. (35) with permission.

**Figure 10:**
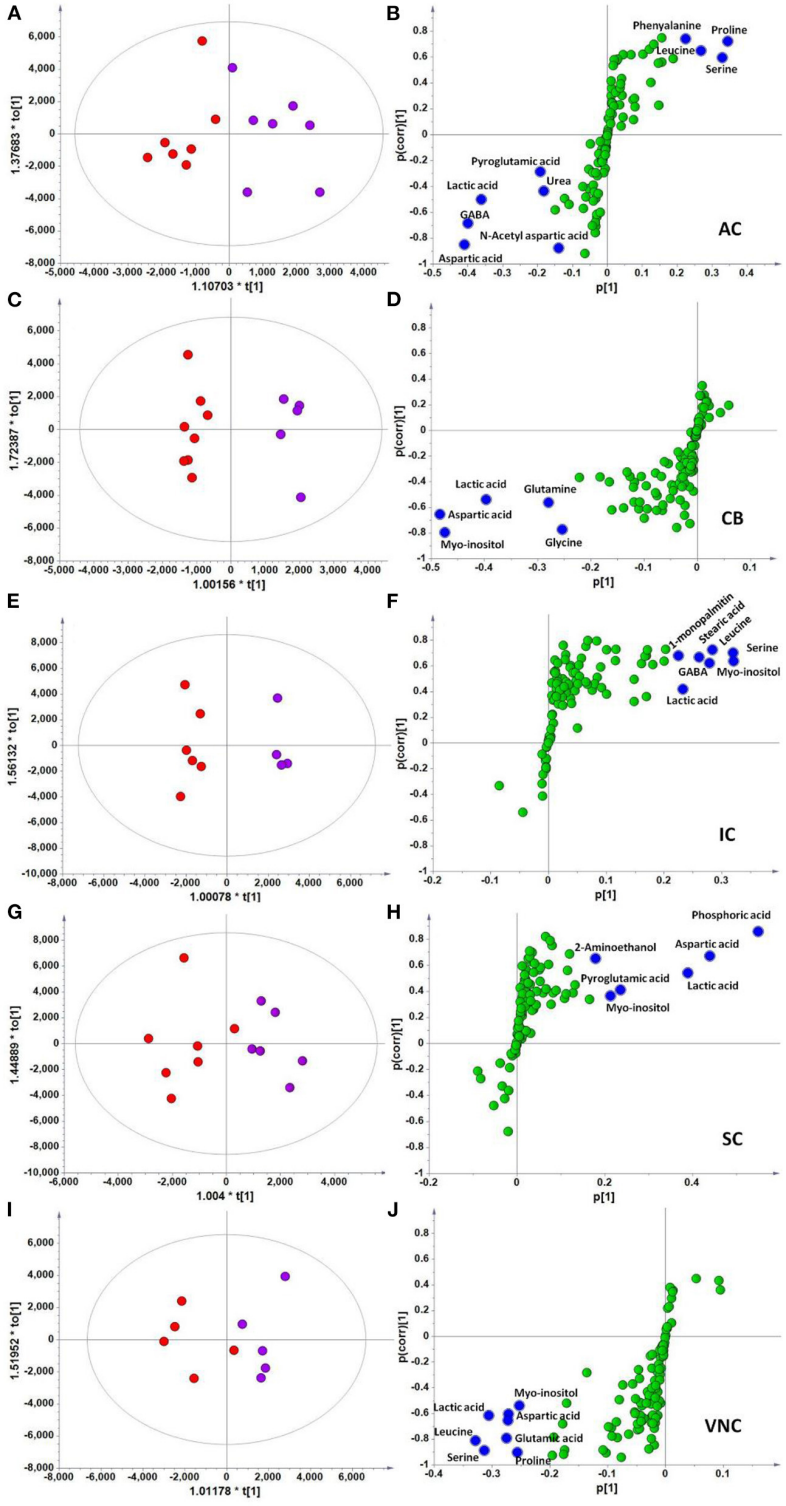
OPLS-DA and S-plot analysis comparing the OPLSDA scores between sham and acoustic trauma animals in different brain regions. Left panel, OPLSDA scores plots, red dots: Sham, purple dots: Acoustic trauma; Right panel, S-plots. **(A,B)** AC [Predictive component: *R2X* = 0.194, *R2Y* = 0.76, *Q*^2^ = 0.45; Orthogonal component 1: *R2X* = 0.446; All components: *R2X* (cum) = 0.64]; **(C,D)** CB [Predictive component: *R2X* = 0.152, *R2Y* = 0.973, *Q*^2^ = 0.68; Orthogonal component 1: *R2X* = 0.364; All components: *R2X* (cum) = 0.927]; **(E,F)** IC [Predictive component: *R2X* = 0.293, *R2Y* = 0.978, *Q*^2^ = 0.702; Orthogonal component 1: *R2X* = 0.417; All components: *R2X* (cum) = 0.905]; **(G,H)** SC [Predictive component: *R2X* = 0.238, *R2Y* = 0.791, *Q*^2^ = 0.691; Orthogonal component: *R2X* = 0.562; All components: *R2X* (cum) = 0.8]; **(I,J)** VNC [Predictive component: *R2X* = 0.403, *R2Y* = 0.779, *Q*^2^ = 0.445; Orthogonal component: *R2X* = 0.389; All components: *R2X* (cum) = 0.792]. In the right panel, the blue dots show variables with high negative magnitude and reliability scores (everything is scaled and relative) or high positive magnitude and reliability scores, i.e., potential biomarkers. AC, auditory cortex; CB, cerebellum; IC, inferior colliculus; CN, cochlear nucleus; VCN, vestibular nucleus complex. From He et al. (35) with permission.

**Figure 11:**
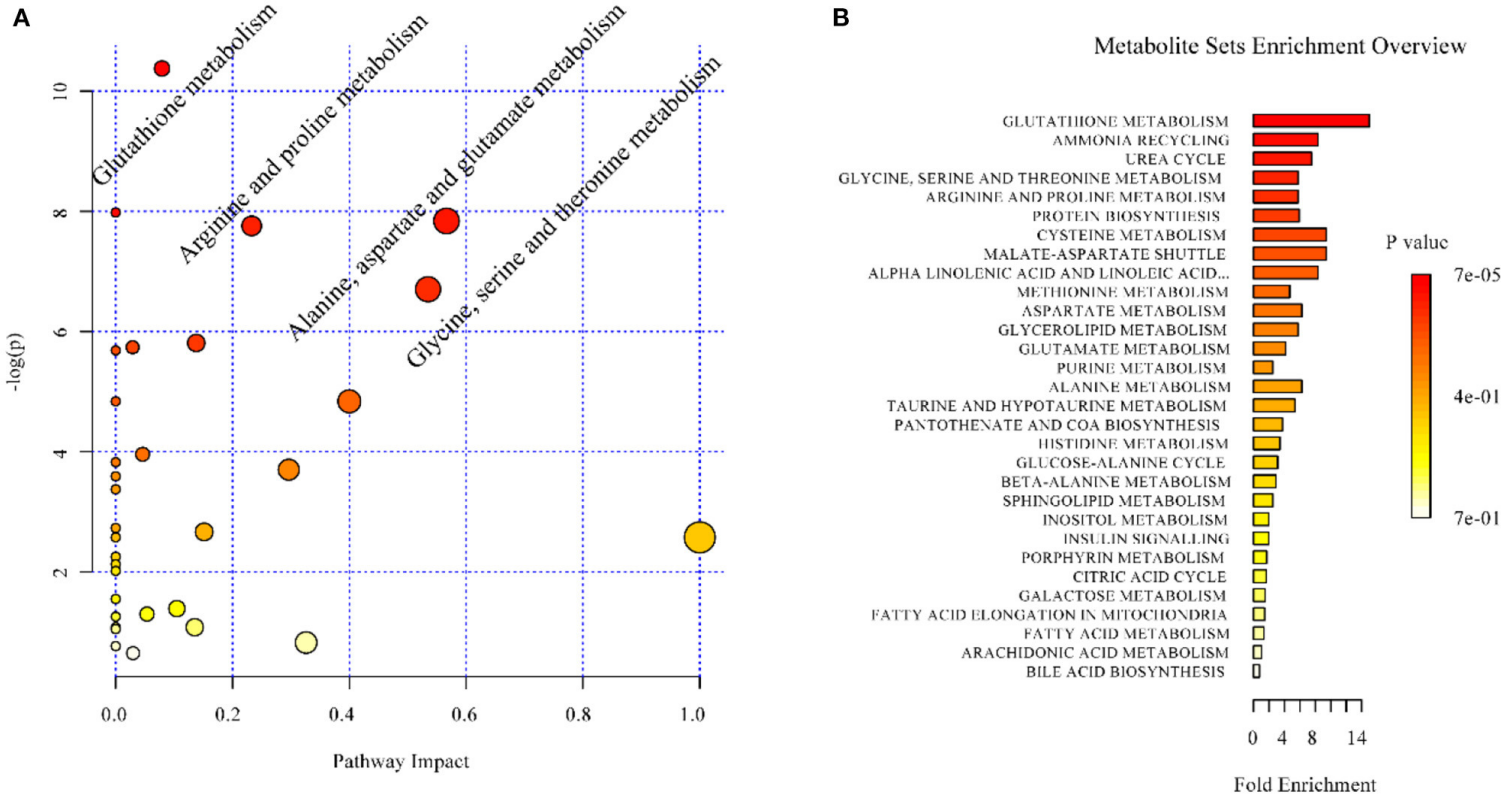
Overview of the impact of acoustic trauma on brain metabolites. **(A)** The pathway impact of acoustic trauma on metabolites. The y axis shows the *p*-values and the x axis, the pathway impact values; the node color is based on its *p*-value and the node size reflects the pathway impact values. **(B)** The enrichment overview of the pathway-associated metabolite sets perturbed by acoustic trauma. From He et al. (35) with permission.

In the context of auditory neuroscience, we have used OPLS-DA on metabolomics data from brain samples to successfully predict whether rats have been exposed to acoustic trauma or a sham procedure (35, 65). The ultimate aim here is to use metabolomic analysis of blood samples to predict whether humans might develop tinnitus or whether they might respond to particular tinnitus treatments (65). In what we believe to be the first study of its kind, we analyzed brain samples from 12 different brain regions in rats that had been exposed to either acoustic trauma or a sham procedure, and used GC-MS to isolate a total of 107 distinct peaks in the chromatogram, with 88 authentically identified as amino acids, small organic acids, carbohydrates, fatty acids, lipids and amines (see Figure 8). PCA and OPLS-DA were performed on the data. In Figure 9, each dot represents the summarized information from the 88 authentically identified molecules for a particular brain region. The distance between the dots indicates the similarity of the metabolic composition of the samples. Brain regions with similar functions appeared to have a similar metabolic composition in both sham and acoustic-trauma exposed animals. However, OPLS-DA in specific brain regions such as the auditory cortex, cerebellum, inferior colliculus, superior colliculus and vestibular nucleus, showed that the metabolic profile was separated for the sham and acoustic-trauma-exposed animals (35). This suggested that a shift in the metabolic pattern had occurred in these brain regions in the animals exposed to acoustic trauma. The associated S plots (Figure 10) indicated that potential biomarkers of acoustic trauma in these brain regions included urea, amino acids, fatty acids, sugar acids, nucleosides and organic acids, in a region-specific fashion. For example, GABA was significantly increased only in the auditory cortex. The overall impact of the acoustic trauma on brain metabolites is summarized in a pathway analysis in Figure 11 (35).

## Summary

Phenomena in vestibular and auditory neuroscience, as in other areas of neuroscience, almost always involve the complex interaction of multiple variables, and yet many areas of basic vestibular and auditory neuroscience, in particular, employ univariate statistical analyses almost exclusively. This may limit the ability of studies to reveal how the interactions between different variables may determine a particular outcome. We have used MVAs and data mining methods to explore the way that combinations of variables can account for neurochemical and behavioral changes following the loss of vestibular function (3–6, 15, 72, 73) and auditory function [e.g., (35, 65)]. In clinical neuroscience research, MVAs and data mining methods have been used to predict the progression of patients from one neurological disorder to another [e.g., (9, 12)] and the probability that the early adolescent use of *Cannabis* can lead to the development of psychotic symptoms in later life [e.g., (74)]. These methods are now in routine use in areas such as genomics, proteomics, metabolomics (10, 11), and the analysis of fMRI data [e.g., (75)]. Electrophysiological research in neuroscience is increasingly moving to the use of multi-electrode arrays using dozens or more micro-electrodes simultaneously, and in this situation one of the main objectives is to determine how different brain regions change in relation to one another, which requires MVA [e.g., (76, 77)].

MVAs and data mining methods can be applied to every aspect of vestibular and auditory neuroscience in order to gain a better understanding of the way in which networks or systems of variables affect otological function. In the search for biomarkers of SNHL, tinnitus and vestibular dysfunction, classification methods such as linear discriminant analysis, support vector machines, random forest classification, Bayesian classifiers and OPLS-DA, can be applied to behavioral, neurophysiological and neurochemical data to predict the probability of a disease or disorder developing, in order to intervene and provide treatments that will prevent or impede the pathological changes. In the context of metabolomics, MVAs and data mining methods have already been proven to be useful in the prediction of disease [e.g., (8, 9, 11–13, 78–80)]. OPLS-DA is an example of an MVA that has successfully been applied to metabolomic data in order to predict hearing loss in rats (35, 65) and may be particularly useful in the search for biomarkers of SNHL, tinnitus, and vestibular dysfunction [e.g., (81)].

## Data Availability Statement

The raw data supporting the conclusions of this article will be made available by the authors, without undue reservation.

## Author Contributions

PS and YZ: conceptualization and writing—review and editing. PS: writing—original draft preparation.

## Conflict of Interest

The authors declare that the research was conducted in the absence of any commercial or financial relationships that could be construed as a potential conflict of interest.

## Abbreviations

GC/MS: gas chromatography/mass spectrometry
MVA: multivariate statistical analysis
OLPSDA: orthogonal partial least squares discriminant analysis
SNHL: sensorineural hearing loss.
